# Isochlorogenic Acid C and *Artemisia argyi* Extract Prevent Acute Gastritis by Mitigating Gastric Mucosa Injury and Mitochondrial Dysfunction

**DOI:** 10.4014/jmb.2511.11015

**Published:** 2025-12-18

**Authors:** So Jeong Paik, Hai-Hua Jiang, Sang-Ho Lee, Eun-Hye Han, Na-Young Yun, Sung Keun Jung

**Affiliations:** 1 School of Food Science and Biotechnology, Kyungpook National University, Daegu 41566, Republic of Korea; 2 Research Center, Dong-A Pharm. Co., Ltd., Yongin 02587, Republic of Korea; 3 Research Institute of Tailored Food Technology, Kyungpook National University, Daegu 41566, Republic of Korea

**Keywords:** Isochlorogenic acid C, gastritis, mitochondria, gastric mucosa, oxidative stress, inflammation

## Abstract

Gastritis is a common inflammatory gastrointestinal disease affecting approximately 40% of the global population. Alcohol abuse is a major contributor to gastritis, primarily acting through gastric mucosal injury and inflammation. Given the association between alcohol consumption and severe gastrointestinal complications, safe and effective strategies for the prevention and treatment of gastritis are urgently needed. This study investigated the pathophysiological effects of ethanol (EtOH) on gastric tissues using an EtOH–HCl-treated acute gastritis mouse model, EtOH-treated AGS gastric epithelial cells, and LPS-treated RAW 264.7 macrophages. We found that EtOH induces acute gastritis by damaging the gastric mucosa, elevating oxidative stress, and disrupting mitochondrial function. We identified Isochlorogenic acid C (ICAC) as a promising therapeutic candidate to counteract these detrimental effects. In the mouse model, oral administration of ICAC significantly alleviated acute gastritis by preserving mucosal integrity, preventing prostaglandin E_2_ depletion, and enhancing *Ptgs1* and *Muc6* expression in mice, while suppressing inflammatory mediator production in gastric tissues. Mechanistically, ICAC’s preventive effects in human cells manifested through *PTGS1* upregulation and the inhibition of inflammatory pathways in AGS cells. Interestingly, ICAC mitigated mitochondrial dysfunction by modulating calcium-mediated mitochondrial fission, reducing mitochondrial reactive oxygen species production, stabilizing mitochondrial membrane potential, and maintaining mitochondrial morphology. Furthermore, the ethanol extract of *Artemisia argyi*, which is composed primarily of ICAC, exhibited comparable protective effects. These findings highlight the detrimental impacts of alcohol on gastric health and identify ICAC as a potential nutraceutical agent for the prevention and treatment of acute gastritis.

## Introduction

Gastritis, an inflammation of the gastric mucosa [1], has a prevalence of 40% in the population [2]. Following the Kyoto global consensus report, gastritis is classified depending on its etiology, accounting for environmental (*e.g.*, alcohol consumption, non-steroidal anti-inflammatory drug exposure, radiation, viral infection, and *Helicobacter pylori* infection) and host-related (*e.g.*, autoimmune response, immune-mediated response, Crohn's disease, and stress) factors [3]. Alcohol consumption, allowing direct contact between alcohol and the mucosa, induces marked mucosal damage, resulting in acute or chronic stomach diseases [4]. Gastritis is classified into acute and chronic gastritis depending on how long the symptoms and signs persist [5]. Alcohol abuse can affect gastric acid secretion, cause acute gastric mucosal injury, and inhibit gastric motility. Even a single instance of heavy alcohol consumption can cause mucosal inflammation and hemorrhagic lesions [4]. Moreover, a clinical study has shown that a history of alcohol abuse markedly increases the risk of severe gastrointestinal events, such as gastrointestinal hemorrhage and/or perforation [6], highlighting the need for effective strategies to prevent and treat alcohol-induced damage and protect gastric health.

Endogenous prostaglandins (PGs) are pivotal in protecting the stomach against mucosal damage and ulceration and stimulating factors crucial for maintaining normal mucosal integrity [7]. Prostaglandins are produced from arachidonic acid, which is synthesized by the enzyme cyclooxygenase (COX) [8]. Prostaglandin E_2_ (PGE_2_) provides mucosal protection and lesion healing primarily by reacting with PGE_2_ receptors. Both COX-1 and COX-2 are related to the roles of PGE_2_: COX-1 mainly produces endogenous PGE_2_ involved in mucosal protection, while COX-2 is important in gastric ulcer healing [9].

Mitochondria are highly dynamic double-membrane organelles that serve as the central hub for cellular metabolism, stress responses, and the maintenance of homeostasis [10]. To maintain these functions, mitochondria must alter their activity to meet cellular needs, balancing their involvement accordingly. In addition to responding to internal and external signals, mitochondria provide signals to the nucleus through multiple mechanisms, including reactive oxygen species (ROS) production and cytochrome C release [11], thereby influencing cellular function [12]. Mitochondrial dysfunction elicits cellular stress responses, and mitochondria-dependent signaling has been shown to contribute to a wide range of physiological and pathophysiological outcomes [13]. Notably, mitochondrial calcium (Ca^2+^) is essential for regulating various cellular functions and maintaining Ca^2+^ signaling, which plays a critical role in preserving homeostasis and preventing disease development [14]. However, excessive mitochondrial Ca^2+^ influx can lead to elevated ROS production, resulting in mitochondrial oxidative stress [15]. Ethanol (EtOH) has been shown to cause gastropathy by disturbing mitochondrial structure and inducing oxidative stress [16], but the precise mechanisms by which mitochondrial disruption contributes to EtOH-induced gastritis have not been fully elucidated.

Isochlorogenic acid C (ICAC) is a caffeoylquinic acid derivative composed of two caffeic acid moieties esterified to a single quinic acid core [17]. Naturally existing in various plant species, ICAC exhibits multiple physiological activities, including anti-cancer [17], anti-influenza virus [18], antihepatotoxic [19], and anti-inflammatory effects [20]. However, its potential to protect against gastritis caused by alcohol has not yet been assessed. Thus, this study aimed to investigate the signaling mechanism underlying EtOH-induced gastritis and evaluate the preventive efficacy of ICAC against this pathological condition. Furthermore, we assessed the gastroprotective effects of a natural plant extract enriched in ICAC to investigate its potential as a therapeutic candidate derived from natural sources.

## Materials and Methods

### Chemicals

Isochlorogenic acid C (CAS No. 57378-72-0, 99.78% purity) was purchased from iKSChem (China) and diluted in dimethyl sulfoxide (Sigma-Aldrich, USA) to create a 50 mM stock solution. Absolute ethyl alcohol (EtOH) and hydrochloric acid (HCl) were obtained from Sigma-Aldrich. Lipopolysaccharide (LPS) derived from *Escherichia coli* O111:B4 (Sigma-Aldrich) was diluted in serum-free Dulbecco's modified Eagle's medium (DMEM; Hyclone, USA) without phenol-red to create a 1 mg/ml stock solution.

### Animal Experiments

Male ICR mice (5-week-old) were acclimated for 7 d under a 12 h light–dark cycle with ad libitum access to a standardized diet and sterilized water. All experimental procedures were conducted in accordance with ethical guidelines and approved by the Institutional Animal Care and Use Committee of Kyungpook National University (KNU-2022-0385).

Following acclimation, mice were orally administered ICAC dissolved in 1% hydroxypropyl methylcellulose or the ethanol extract of *Artemisia argyi* (EAA) once daily for three consecutive days. After the final administration, food was withdrawn for 24 h. The mice were then re-administered ICAC or EAA, followed, after 1 h, by the oral administration of 150 mM HCl in 70% EtOH to induce acute gastritis. One hour later, gastric tissues were harvested for analysis.

### H&E Staining

Hematoxylin and eosin staining was used to evaluate structural damage. Gastric tissues were soaked in 10%neutral buffered formalin solution (Sigma-Aldrich) and embedded in paraffin blocks. Tissue sections (5 μm) were cut from the paraffin blocks, dipped in xylene (OCI Company Ltd., Republic of Korea), and soaked in 95% ethanol. Tissue fixation was performed using 4% formaldehyde, and then hematoxylin solution (TissuePro Technology LLC.; USA) was used for nuclear staining. Subsequently, tissues were counterstained using Eosin Y (TissuePro Technology LLC.). After dipping in 95% and 100% ethanol, tissues were soaked with xylene, coverslipped with a mounting medium, and dried. Histological observations were recorded using a microscope (Leica Microsystems, Germany) with Leica Application Suite V4 (Leica Microsystems).

### PGE_2_ Measurement

Gastric tissues were treated with a lysis buffer (Cell Signaling Technology, USA) containing a protease inhibitor cocktail (Thermo Scientific, USA). The mixture was centrifuged, and the supernatant was collected for analysis. We measured PGE_2_ using a PGE_2_ ELISA assay kit (KGE004B; R&D Systems, USA) following the manufacturer’s guidelines.

### Cell Cultures

Human gastric epithelial cell line AGS cells (KCLB No. 21739; Korean Cell Line Bank, Republic of Korea) were cultured in RPMI-1640 medium supplemented with fetal bovine serum (FBS) at 10% and penicillin-streptomycin at 1%. Murine macrophage cell line RAW 264.7 cells (KCLB No. 40071, Korean Cell Line Bank) were maintained in DMEM supplemented with FBS at 10% and penicillin-streptomycin at 1%. All cultures were incubated at 37°C in a humidified atmosphere containing 5% CO_2_.

### qRT-PCR Analyses

Quantitative real-time reverse transcription PCR (qRT-PCR) processes followed the protocols provided by suppliers. For mouse gastric tissues, shredded tissue (*n* = 5) was pulverized in RNA isolation buffer with beads using a homogenizer at 6,000 rpm in 2 runs with 3 cycles each. The tissue was incubated in chloroform and centrifuged at 12,000 ×*g* and 4°C for 15 min. The RNA layer was collected and mixed with isopropyl alcohol, and the precipitate was washed in 75% ethanol. The dried pellet was then diluted in DEPC water, and RNA was quantified using a nanodrop spectrophotometer. Subsequently, RT Master Mix (Toyobo, Japan) was mixed with the purified RNA to synthesize the cDNA, which was used as the template for qRT-PCRs. Reaction mixtures contained SYBR Green (Toyobo) and the appropriate primers for the gene of interest (Table S1), and relative expression was calculated using the ΔΔCq method.

For cell culture expression analyses, AGS cells (2 × 10^5^ cells/ml) were seeded into 6-cm dishes and grown until reaching 70% confluence. Cells were then treated with EtOH for 15 min, 30 min, or 1 h to assess the temporal dynamics of the prostaglandin-endoperoxide synthase 1 (*PTGS1*) gene expression response. To assess the preventive effect of ICAC against EtOH-induced *PTGS1* suppression, cells were incubated in ICAC for 1 h and then stimulated by EtOH for 1 h. Subsequent extraction and qRT-PCR procedures were performed using the protocols described above for stomach tissue.

### ELISA

Pro-inflammatory cytokine secretion in gastric tissues was assessed using an enzyme-linked immunosorbent assay (ELISA; Thermo Scientific) kits. Shredded tissue was crushed in lysis buffer containing a protease inhibitor, and the supernatant was obtained. The ELISA assay was then performed according to the manufacturer’s protocol. Briefly, the wells of 96-well plates were coated with capture antibody using the coating buffer. After incubating overnight, the plates were blocked with ELISA diluent. Tissue supernatants and standards were added, and the plates were incubated for 2 h. The detection antibody was then added, followed by incubation, and then horseradish peroxidase was applied. Lastly, the 3, 3', 5, 5'-tetramethylbenzidine solution and then the stop solution were added. Absorbance was measured at wavelengths of 450 and 570 nm.

### Nitrite Assays

To quantify nitrite production in gastric tissue, tissues were ground in lysis buffer containing a protease inhibitor, and the supernatant was obtained. A nitrite assay was performed as described in our previous study [21]. Briefly, the supernatants were reacted with a mixture of Griess reagents A and B for 30 min. Absorbance at 550 nm was detected using a microplate reader (SpectraMax iD3; Molecular Devices, USA). To assess nitrite production in human gastric epithelial cells *in vitro*, AGS cells (5 × 10^5^ cells/ml) were grown in 96-well plates overnight and then incubated with ICAC for 24 h. EtOH was added after 1 h of ICAC treatment. Nitrite levels in the AGS cells were measured using the procedures described for gastric tissues.

### GPx Activity Measurement

Glutathione peroxidase (GPx) activity was measured using the Glutathione Peroxidase Assay Kit (Abcam, UK) according to the manufacturer’s instructions. Briefly, 30 mg of gastric tissue was homogenized in assay buffer, and the supernatant was collected after centrifugation at 10,000 ×*g* for 15 min. To eliminate glutathione disulfide, the supernatant was incubated with 40 mM NADPH, active glutathione reductase, and glutathione for 15 min. To quantify GPx activity, cumene hydroperoxide was added to start the GPx reaction, the solution’s absorbance at 340 nm was measured at two different time points, and GPx activity was calculated based on the rate of NADPH oxidation.

### Western Blots

Cell contents were extracted in a lysis buffer containing phosphatase and protease inhibitors, and then the mixture was centrifuged to obtain the cell lysates. Proteins were then quantified using a DC protein assay kit (Bio-Rad Inc., USA). Subsequently, the proteins were separated through electrophoresis using a 10% sodium sulfate-polyacrylamide gel and transferred to 0.45 μm Immobilon polyvinyl fluoride membranes (Millipore, USA). After blocking with 5% skim milk, the membranes were incubated overnight with primary antibodies against COX-2, p-DRP1 (Ser616), DRP1, MCU (Cell Signaling), NOS2, β-actin (Santa Cruz Biotechnology, USA), and α/β-tubulin (Abcam) and then secondary antibodies. Proteins were stained using the EzWestLumi Plus Kit (ATTO, USA) and imaged using a GeneGnome XPQ NPC system (Syngene, UK).

### Cytotoxicity Assessment

AGS cells were seeded into 96-well plates at 2 × 10^5^ cells/ml and grown until reaching 60–70% confluency. The cells were then incubated with ICAC for 24 h. Cell viability was measured using the protocols described in our previous study [21]. In brief, cells were incubated in a 3-(4,5-dimethylthiazol-2-yl)-2,5-diphenyltetrazolium bromide solution for 2 h. Then, dimethyl sulfoxide was added, and the plates were incubated for 30 min in a dark room. Cell survival rate was measured as the absorbance at 570 nm using a microplate reader (SpectraMax iD3). The cytotoxicity of ICAC against RAW cells was also assessed using the same procedures, except RAW cells were originally seeded at 3 × 10^5^ cells/ml.

### Mitochondrial Localization Assessment

AGS and RAW 264.7 cells were seeded in triplicate into 48-well plates at 1 × 10^5^ and 2 × 10^5^ cells/ml, respectively, and the plates were incubated overnight. To evaluate the temporal dynamics of EtOH and LPS’s effects on mitochondrial localization, cells were grown with 2% EtOH or 1 μg/ml LPS for 1, 3, and 6 h. To assess the protective effects of ICAC, cells were incubated with ICAC for 1 h and then with EtOH or LPS for 6 h. For all treatments, cells were stained in 50 nM (for AGS cells) or 100 nM (for RAW 264.7 cells) Mito-tracker Green FM (M7514; Molecular Probes, USA) solutions for 15 min. Mitochondria were observed using a fluorescence microscope (Leica Microsystems) and imaged using Leica Application Suite X (Leica Microsystems).

### Mitochondrial Membrane Potential Assessment

AGS cells were seeded at 1 × 10^5^ cells/mL and grown in 8-well chambers (Ibidi, USA) and incubated overnight. To evaluate the temporal dynamics of EtOH’s effects on mitochondrial membrane potential (ΔΨ_M_), cells were treated with EtOH for 1, 3, and 6 h. To evaluate the protective effect of ICAC, cells were pre-treated with ICAC for 1 h and subsequently treated with EtOH for 1 h. For all treatments, mitochondrial membranes were stained with JC-1 dye (2 μM) for 15 min, nuclei were stained with mounting medium (Abcam), and the cells were visualized using a fluorescence microscope (Leica Microsystems).

For RAW 264.7 cells, the evaluation of ΔΨ_M_ was carried out following previously reported procedures [22]. RAW cells were seeded at 2 × 10^5^ cells/ml into 8-well chambers (Ibidi) and grown until reaching 60% confluency. Cells were pre-treated with ICAC for 1 h and treated with LPS for 3 h. Next, the cells were incubated with JC-1 dye (1 μg/ml) for 15 min, and cell nuclei were counterstained with mounting medium (Abcam). The cells were then visualized using a fluorescence microscope (Leica Microsystems).

### Intracellular ROS Measurement

AGS cells were seeded into 96-well plates at 2 × 10^5^ cells/ml and allowed to grow overnight. To evaluate the effect of EtOH on intracellular ROS production over time, cells were treated with EtOH for 3, 6, 12, and 24 h. To evaluate the effect of ICAC on EtOH-induced ROS production, cells were first incubated with ICAC for 1 h and then with EtOH for 6 h. For all treatments, the cells were stained in a 2',7'-dichlorofluorescein diacetate (DCF-DA) solution for 30 min. Intracellular ROS was then quantified using a microplate reader (SpectraMax iD3), and fluorescence images were obtained using a fluorescence microscope (Leica Microsystems) with Leica Application Suite X (Leica Microsystems).

RAW 264.7 cells were seeded at 3 × 10^5^ cells/ml into 96-well plates, and the plates were incubated until reaching 60–70% confluency. Cells were treated with ICAC for 1 h, followed by LPS stimulation for 24 h. Intracellular ROS was measured using the procedures described for AGS cells above.

### mtROS Assessment

AGS cells were seeded into 8-well chambers (Ibidi) at 1 × 10^5^ cells/ml and allowed to grow overnight. To evaluate the temporal dynamics of EtOH’s effects on mitochondrial ROS (mtROS) production, cells were treated with EtOH for 30 min, 1 h, and 3 h, respectively. To evaluate the protective efficacy of ICAC, cells were pre-treated with ICAC for 1 h and treated with EtOH for 1 h. For both experiments, the cells were washed with Hanks’ balanced salt solution (HBSS) and stained in a 2 μM MitoSOX Red (M26008; Invitrogen, USA) solution for 10 min. The cells were fixed with 4% paraformaldehyde (Sigma-Aldrich) and counter-stained with mounting medium with DAPI (Abcam) to stain cell nuclei. Visualization of mtROS was performed using a fluorescence microscope (Leica Microsystems) with Leica Application Suite X (Leica Microsystems).

RAW cells were seeded into 8-well chambers (Ibidi) and allowed to grow until reaching 60–70% confluency. Cells were treated with ICAC and then LPS for 1 h each. Procedures for staining and observing mtROS were performed as described above for AGS cells.

### Mitochondrial Ca^2+^ Assessment

AGS cells were seeded at 1.5 × 10^5^ cells/ml into 48-well plates, and the plates were incubated overnight. To evaluate the effects of EtOH on mitochondrial Ca^2+^ over time, cells were stimulated with EtOH for 30 min and 1 h. To assess the effect of ICAC on EtOH-induced Ca^2+^ influx, cells were pre-treated with ICAC for 1 h and then treated with EtOH for 30 min. For all treatments, cells were stained in a 50 nM Mito-tracker Green FM (M7514; Molecular Probes) solution for 15 min. Cells were further stained in a 5 μM Rhod-2 AM (HY-D0989; MedChemExpress, USA) solution for 15 min. Mitochondrial Ca^2+^ was observed using a fluorescence microscope (Leica Microsystems) with Leica Application Suite X (Leica Microsystems).

### *Artemisia argyi* Extract

To confirm the effect of a botanical material known to primarily be composed of ICAC, EAA was obtained from Dong-A Pharm (Republic of Korea).

## Results

### Oral Administration of ICAC Alleviates Acute Gastritis in Mice

**Fig. 1:**
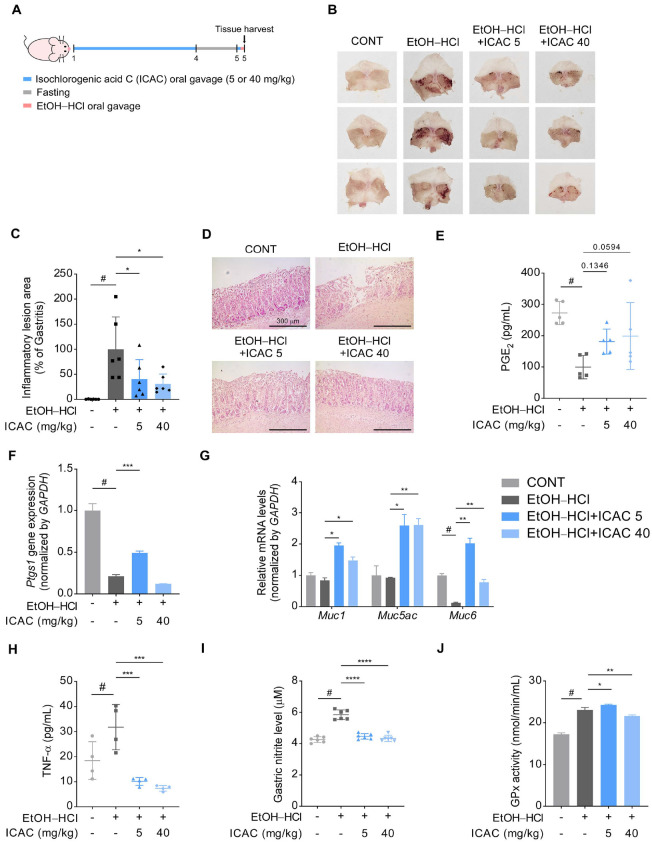
Oral administration of Isochlorogenic acid C (ICAC) alleviates acute gastritis in an EtOH–HCltreated mouse model. (**A**) A schematic illustrating the experimental protocol. Mice were orally administered ICAC daily for three consecutive days. Then, after 24 h of fasting, the mice received an additional dose of ICAC followed by EtOH–HCl administration. (**B**) Representative images of harvested mouse stomachs. (**C**) The proportion of mucosal area exhibiting inflammatory lesions in the mouse stomachs (*n* = 6). Data are presented as means ± the standard deviation (SD). (**D**) Representative histological images of mouse stomach tissue sections stained with hematoxylin and eosin (10× objective; scale bars = 300 μm). (**E**) PGE_2_ concentrations in mouse stomach tissue, as measured by ELISAs (*n* = 5). Data are presented as means ± SD. (**F**) *Ptgs1* and (**G**) *Muc1*, *Muc5ac*, and *Muc6* expression levels, assessed using qRT-PCR (*n* = 4). Data are presented as means ± the standard error of the mean (SEM). (**H**) TNF-α secretion in mouse stomach tissue measured using ELISAs (*n* = 4). Data are shown as means ± SD. (**I**) Nitrite levels in mouse stomach tissue, analyzed using the Griess assay (*n* = 6). Data are shown as means ± SD. (**J**) Glutathione peroxidase (GPx) activity in the stomach tissue of mice, measured using the GPx assay (*n* = 5). Data are expressed as means ± SD. Statistical significance was analyzed using one-way ANOVAs followed by Dunnett’s *post hoc* tests. Significance levels are represented by symbols: #, *p* < 0.05, for comparisons between the EtOH–HCl only-treated and non-treated groups and *, *p* < 0.05; **, *p* < 0.01; ***, *p* < 0.001; and ****, *p* < 0.0001, for comparisons between the ICACtreated groups and the EtOH–HCl only-treated group.

To explore the preventive effect of ICAC on acute gastritis, mice were orally administered ICAC for 3 d, fasted for 24 h, orally administered ICAC, and then, 1 h later, orally administered an EtOH–HCl mixture (Fig. 1A). Mouse stomachs were subsequently harvested and analyzed. While the EtOH–HCl treatment induced inflammation in the stomach lining, ICAC prevented EtOH–HCl-induced gastritis in a dose-dependent manner (Fig. 1B and 1C). As shown in Fig. 1D, gastric tissue structure and the mucus layer were disrupted by EtOH–HCl; however, ICAC greatly alleviated these pathological effects.

Homeostasis of the gastric mucus layer contributes crucially to stomach health, and PGE_2_ is involved in maintaining this layer [7]. Although EtOH reduced PGE_2_ secretion in gastric tissues, ICAC effectively attenuated this PGE_2_ reduction (Fig. 1E). The human *PTGS1* gene encodes COX-1, which is directly involved in PGE_2_ synthesis [23], and ICAC reduced *Ptgs1* downregulation in mice when administrated at 5 mg/kg, but not at 40 mg/kg (Fig. 1F). In stomach tissue, mucin secretion is also important in maintaining the gastric mucus layer, and in normal gastric mucosa cells, the mucin-encoding genes *Muc1*, *Muc5ac*, and *Muc6* are highly expressed [24]. Based on this knowledge, we investigated the effect of ICAC on mucin secretion-related genes under EtOH–HCl-induced mucosa inflammation. The expression of *Muc6* was significantly reduced by EtOH–HCl, while *Muc1* and *Muc5ac* expression did not exhibit any changes in response to the EtOH–HCl treatment. Pretreatment with ICAC increased *Muc6* expression in EtOH–HCl-treated mice (Fig. 1G). Moreover, the ICAC pretreatment reduced EtOH–HCl-induced TNF-α secretion and nitrite production to at or below normal levels (Fig. 1H and 1I). Furthermore, at 40 mg/kg, ICAC suppressed GPx activity, counteracting EtOH–HCl-induced increases (Fig. 1J).

### ICAC Exerts Homologous Protective Effects against EtOH in Human Gastric System Cells

**Fig. 2:**
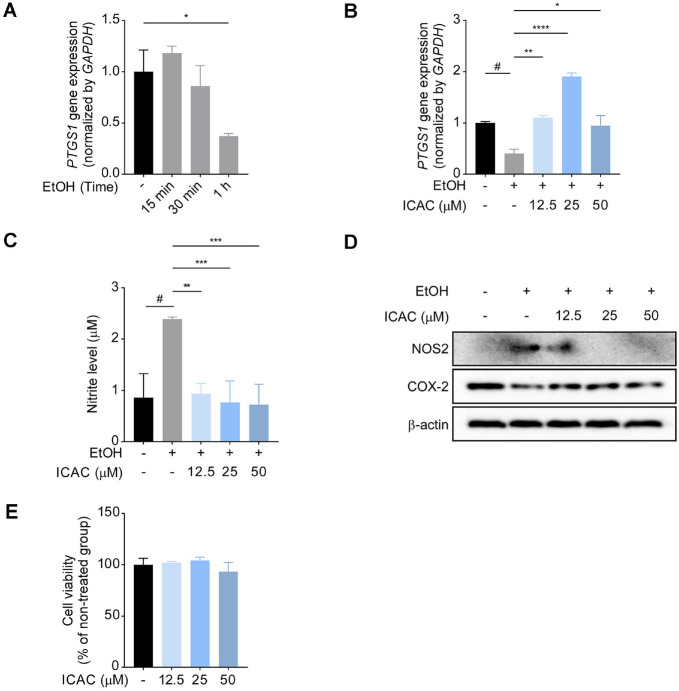
ICAC exerts homologous protective effects against EtOH in AGS cells. (**A**) Time-dependent alterations to *PTGS1* expression following EtOH exposure. (**B**) The effect of ICAC pretreatment on *PTGS1* expression after 1 h of EtOH exposure, analyzed using ELISA. Data are presented as means ± SEM. (**C**) The effect of ICAC pretreatment on nitrite production after 24 h of EtOH exposure, quantified using the Griess assay. Data are presented as means ± SD. (**D**) A Western blot membrane image illustrating the effect of ICAC on NOS2 and COX-2 expression after 6 h of EtOH exposure. (**E**) The cell viability of ICAC-treated AGS cells, measured using the MTT assay. Data are shown as means ± SD. All procedures were performed in triplicate. Significance levels are represented by symbols: #, *p* < 0.05, for comparisons between the EtOH-onlytreated and non-treated groups and *, *p* < 0.05; **, *p* < 0.01; ***, *p* < 0.001; and ****, *p* < 0.0001, for comparisons between the ICAC-treated groups and the EtOH-only-treated group.

In line with the preventive effects of ICAC demonstrated in the animal experiments, we validated the homologous effects of ICAC in human AGS cells *in vitro*, assessing its ability to protect against EtOH toxicity. We selected 2% EtOH as a treatment condition, as it did not induce cell death in AGS cells during pretrials (Fig. S1). To validate the protective effect of ICAC on gastric mucosal injury, we first evaluated *PTGS1* expression changes during EtOH treatment over time. The 2% EtOH treatment significantly downregulated *PTGS1* after 1 h of treatment (Fig. 2A); however, pretreatment with ICAC suppressed EtOH-induced *PTGS1* downregulation (Fig. 2B). Next, we investigated whether ICAC inhibits nitrite production in AGS cells, as it did in mouse stomach tissue. The ICAC pretreatment alleviated EtOH-induced nitrite production (Fig. 2C) and, furthermore, suppressed the expression of NOS2, a protein involved in nitrite production. Additionally, ICAC inhibited the EtOH-induced downregulation of COX-2, another gene directly involved in PGE_2_ synthesis (Fig. 2D). Notably, ICAC did not exhibit cytotoxicity at any tested concentration (Fig. 2E).

### ICAC Prevents Gastritis by Counteracting Mitochondrial Dysfunction

**Fig. 3:**
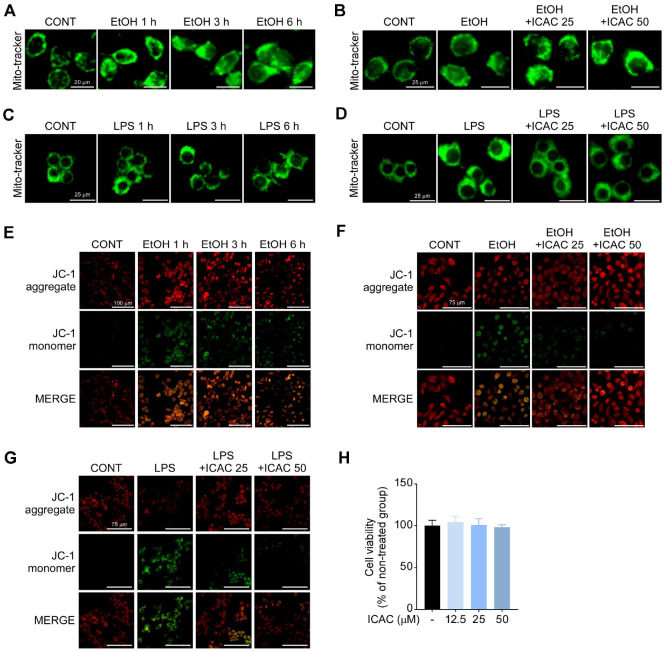
ICAC prevents gastritis by counteracting EtOH- or lipopolysaccharide (LPS)-induced mitochondrial dysfunction. (**A**) Time-dependent changes in mitochondrial morphology in AGS cells following EtOH exposure, visualized using Mito-tracker Green FM (40× objective; scale bars = 20 μm). (**B**) The effect of ICAC on mitochondrial morphological changes in AGS cells after 6 h of EtOH exposure (40× objective; scale bars = 25 μm). (**C**) Time-dependent changes in mitochondrial morphology in RAW 264.7 cells following LPS exposure. Cells were visualized using Mito-tracker Green FM (40× objective; scale bars = 25 μm). (**D**) The effect of ICAC on mitochondrial morphological changes in RAW 264.7 cells 6 h after LPS exposure (40× objectives; scale bars = 25 μm). (**E**) Time-dependent alterations to mitochondrial membrane potential (ΔΨ_M_) in AGS cells following EtOH exposure, analyzed using JC-1 staining (10× objective; scale bars = 100 μm). (**F**) The effect of ICAC on ΔΨ_M_ loss 1 h after EtOH exposure in AGS cells (10× objective; scale bars = 75 μm). (**G**) The effect of ICAC on ΔΨ_M_ loss 3 h after LPS exposure in RAW 264.7 cells (10× objective; scale bars = 75 μm). (**H**) The cell viability of RAW 264.7 cells treated with various test concentrations of ICAC, determined using the MTT assay. Data are presented as the mean ± SD of three replicates.

Mitochondria can be damaged by an imbalance of gastric health; therefore, we investigated whether EtOH induces mitochondrial dysfunction, and whether ICAC can prevent this phenomenon. Initially, we assessed mitochondrial localization following EtOH treatment, and the pattern of mitochondrial morphology was altered over time in AGS cells (Fig. 3A). However, ICAC inhibited EtOH’s effects on mitochondrial morphology (Fig. 3B). Similarly, LPS altered mitochondrial morphology over time in RAW 264.7 cells (Fig. 3C), while ICAC suppressed these changes (Fig. 3D). Next, we stained AGS cells with the JC-1 probe to elucidate the effects of the treatments on ΔΨ_M_. Monomers of JC-1 increased under the EtOH treatment over time (Fig. 3E), and ICAC reduced EtOH-induced JC-1 monomer formation (Fig. 3F). We also confirmed that ICAC prevented JC-1 monomer formation under LPS stimulation in RAW 264.7 cells (Fig. 3G). As with AGS cells, ICAC did not exhibit cytotoxicity against RAW 264.7 cells at any tested concentration (Fig. 3H).

### ICAC Mitigates Mitochondrial Dysfunction by Alleviating Oxidative Stress

**Fig. 4:**
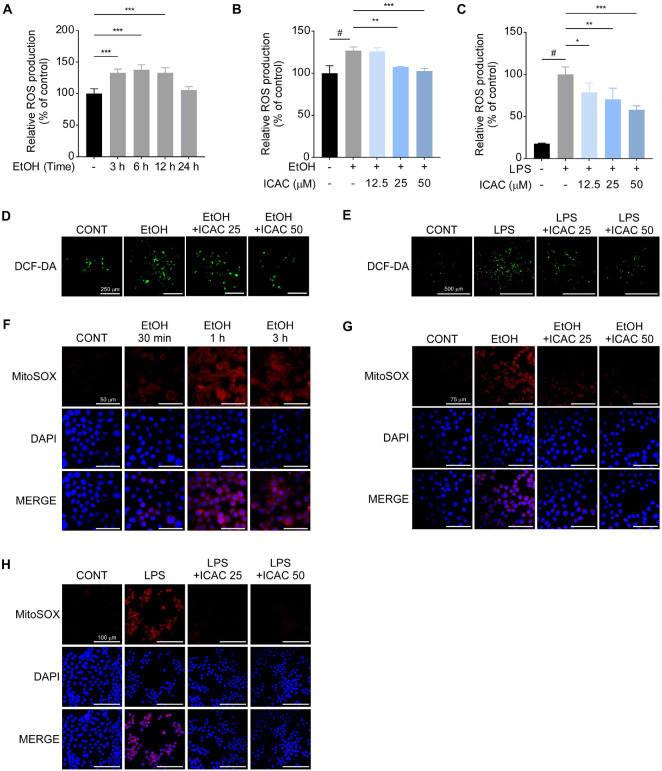
ICAC mitigates EtOH- or LPS-induced mitochondrial dysfunction by alleviating oxidative stress. (**A**) Time-dependent changes in intracellular reactive oxygen species (ROS) production in AGS cells following EtOH exposure, assessed using DCF-DA probes. (**B**) The effect of ICAC on ROS overproduction in AGS cells 6 h after EtOH exposure. (**C**) The effect of ICAC on ROS overproduction in RAW 264.7 cells 24 h after LPS treatment. Data are presented as means ± SD. Representative fluorescence images showing the effect of ICAC on ROS overproduction in (**D**) AGS cells following EtOH exposure (10× objective; scale bars = 250 μm) and (**E**) RAW 264.7 cells following LPS exposure (10× objective; scale bars = 500 μm). (**F**) Time-dependent changes in mitochondrial ROS (mtROS) levels in AGS cells following EtOH treatment, visualized using MitoSOX Red staining and DAPI counterstaining (20× objective; scale bars = 50 μm). The effects of ICAC on mtROS production in (**G**) AGS cells 1 h after EtOH exposure (20× objectives scale bars = 75 μm) and (**H**) RAW 264.7 cells 1 h after LPS exposure (20× objective; scale bars = 100 μm). All experiments were performed in triplicate. Significance levels are represented by symbols: #, *p* < 0.05, for comparisons between the EtOH/LPS only-treated and non-treated groups and *, *p* < 0.05; **, *p* < 0.01; and ***, *p* < 0.001, for comparisons between the ICAC-treated groups and the EtOH/LPS only-treated group.

Stress can stimulate ROS production in mitochondria, which can, in turn, result in ROS stress [13]. To demonstrate the effect of ICAC on intracellular ROS production, we initially investigated the temporal dynamics of EtOH’s effects on intracellular ROS levels. In AGS cells, EtOH elevated ROS production until 6 h after treatment, but levels then plateaued until 12 h and returned to normal levels by 24 h (Fig. 4A). Thus, we investigated the effects of ICAC pretreatments on EtOH-induced ROS overproduction after 6 h of EtOH treatment, and ICAC suppressed ROS overproduction caused by EtOH in a dose-dependent manner (Fig. 4B). Similarly, ICAC inhibited LPS-induced ROS overproduction in a dose-dependent manner in RAW 264.7 cells (Fig. 4C). The preventive effects of ICAC on ROS production were also observed using fluorescence microscopy in both AGS and RAW 264.7 cells (Fig. 4D and 4E).

Next, we assessed the influence of EtOH on mtROS, first assessing the effects of EtOH alone over time. Although mtROS production was most severely increased after 1 h of treatment with EtOH (Fig. 4F), ICAC markedly alleviated EtOH-induced mtROS overproduction (Fig. 4G). In RAW 264.7 cells, ICAC exhibited similar protective effects against mtROS overproduction induced by LPS (Fig. 4H).

### ICAC Prevents Calcium Influx-Mediated Mitochondrial Fission

**Fig. 5:**
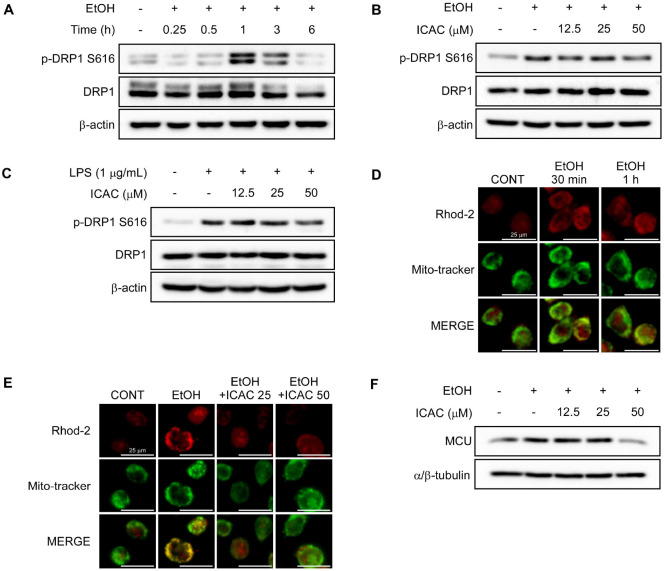
ICAC prevents calcium influx-mediated mitochondrial fission. Western blot membrane images showing (**A**) time-dependent changes in dynamin-related protein 1 (DRP1) phosphorylation at S616 in AGS cells following EtOH exposure, (**B**) the effect of ICAC on DRP1 S616 phosphorylation in AGS cells 1 h after EtOH exposure, and (**C**) the effect of ICAC on DRP1 S616 phosphorylation in RAW 264.7 cells 30 min after LPS treatment. (**D**) Time-dependent changes in mitochondrial calcium (Ca^2+^) localization in AGS cells following EtOH exposure, visualized using Rhod-2 AM probes (40× objective; scale bars = 25 μm). (**E**) The effect of ICAC on Ca^2+^ accumulation in AGS cells 30 min after EtOH exposure (40× objective; scale bars = 25 μm). (**F**) A western blot membrane image showing the effect of ICAC on mitochondrial calcium uniporter (MCU) expression in AGS cells 15 min after EtOH exposure.

Since mitochondrial and cellular ROS levels are directly influenced by alterations in the mitochondrial fission–fusion cycle [25], we investigated the temporal dynamics of DRP1 phosphorylation at Ser616 residue during EtOH treatment. In AGS cells, exposure to EtOH increased phosphorylation after 1 h of exposure, but ICAC attenuated this EtOH-induced phosphorylation (Fig. 5A and 5B). Moreover, ICAC also suppressed DRP1 phosphorylation at Ser616 in response to LPS stimulation in RAW 264.7 cells (Fig. 5C).

Given that mtROS overproduction can be triggered by excessive mitochondrial calcium influx [15], we assessed the impact of EtOH on mitochondrial Ca^2+^ uptake in AGS cells and investigated whether ICAC could prevent EtOH-induced mitochondrial Ca^2+^ overload using Rhod-2 AM, a mitochondrial calcium indicator [26]. Mitochondrial Ca^2+^ accumulation peaked after 30 min of EtOH exposure (Fig. 5D), and ICAC prevented this EtOH-induced influx (Fig. 5E). To investigate this Ca^2+^ influx further, we assessed whether it was mediated by mitochondrial Ca^2+^ transporters, examining the expression of mitochondrial calcium uniporter (MCU). The EtOH treatment significantly increased MCU expression within 15 min of exposure, but ICAC suppressed MCU upregulation (Fig. 5F).

### Oral Administration of EAA, Consisting Primarily of ICAC, Alleviates Acute Gastritis

**Fig. 6:**
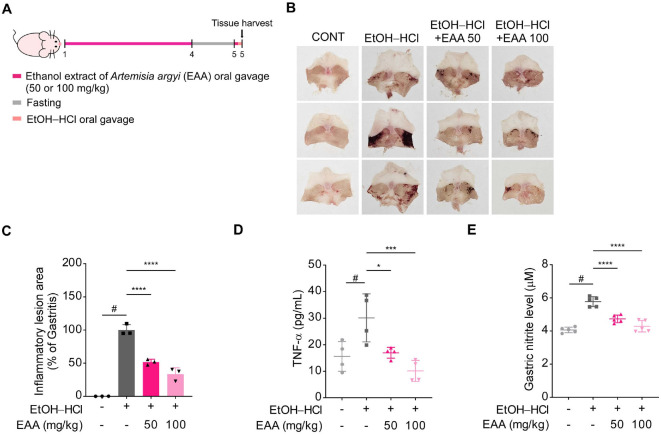
Oral administration of an ethanol extract of *Artemisia argyi* (EAA), which contains ICAC as a primary constituent, alleviates acute gastritis in an EtOH–HCl-treated mouse model. (**A**) A schematic illustrating the experimental protocol. Mice were orally administered EAA daily for three consecutive days. Then, after 24 h of fasting, the mice received an additional dose of EAA, followed by EtOH–HCl administration. (**B**) Representative images of harvested mouse stomachs. (**C**) The proportion of mucosal area exhibiting inflammatory lesions in the mouse stomachs (*n* = 3). (**D**) TNF-α secretion in mouse stomach tissue, measured using ELISAs (*n* = 4). (**E**) Nitrite levels in mouse stomach tissue, analyzed using the Griess assay (*n* = 5). Data are presented as means ± SD. Statistical significance was analyzed using one-way ANOVAs followed by Dunnett’s *post hoc* tests. Significance levels are represented by symbols: #, *p* < 0.05, for comparisons between the EtOH–HCl only-treated and non-treated groups and *, *p* < 0.05; ***, *p* < 0.001; and ****, *p* < 0.0001, for comparisons between the ICAC-treated groups and the EtOH–HCl only-treated group.

Having proved that ICAC prevents acute gastritis *in vivo* and *in vitro*, we aimed to prove that an ICAC-rich plant extract can be a promising therapeutic agent. To this end, we obtained EAA, a plant extract containing ICAC as a primary constituent. We orally administered EAA to the mice for 3 d and subsequently removed access to their food (Fig. 6A). After 24 h of fasting, mice were again administered EAA, and 1 h later, an EtOH–HCl mixture was orally gavaged. After 1 h, mice were sacrificed to analyze gastric alterations. The EAA treatment markedly reduced acute gastritis symptoms (Fig. 6B and 6C) and prevented inflammatory responses by suppressing TNF-α secretion and nitrite overproduction (Fig. 6D and 6E). Thus, ICAC-rich natural products have potential as pharmaceuticals for gastritis therapy.

## Discussion

In this study, we aimed to elucidate the signaling mechanisms involved in EtOH-induced gastritis and evaluate the preventive potential of ICAC. To achieve this, we developed an acute gastritis mouse model using ICR mice to assess the gastroprotective effects of orally administered ICAC. Mice were orally administered the test material for three consecutive days. Then, following a 24-h fasting period to ensure an empty stomach, the mice received a final oral dose of the test material and were subsequently challenged with EtOH–HCl by oral gavage. This model clearly demonstrated both the injurious effects of EtOH and the protective efficacy of the administered samples. Notably, EtOH exposure led to the reduced expression of *Ptgs1* in mouse gastric tissues.

To explore the homologous effect and molecular mechanisms underlying this phenomenon in human cells, we established an *in vitro* model using AGS cells and 2% EtOH, a concentration that did not induce cell death. In this human cell model, EtOH decreased *PTGS1* expression, showing consistency with the *in vivo* findings. To our knowledge, most previous studies have focused on phenotypic changes to evaluate therapeutic efficacy, but our approach emphasizes molecular signaling changes. This focus on mechanistic aspects may help identify new molecular targets for the development of gastritis therapies.

Among genes encoding mucins, *Muc6* expression was significantly diminished under conditions mimicking acute gastritis. Under normal gastric mucosa conditions, *Muc1*, *Muc5ac*, and *Muc6* are generally highly expressed, with *Muc6* primarily localized to deep gastric glands. In contrast, *Muc1* and *Muc5ac* are expressed more broadly across superficial epithelia and other epithelial tissues [24]. The localized expression of *Muc6* underscores its specific role in maintaining gastric mucosal integrity, particularly during the onset of acute gastritis.

Alcohol-induced gastritis was also characterized by disrupted mitochondrial structure and increased oxidative stress. Although Pan *et al*. [16] previously suggested that disruptions in mitochondrial energy metabolism may contribute to ethanol-related gastropathy, their study did not specifically address ΔΨ_M_. We showed that EtOH reduced ΔΨ_M_ in AGS cells and that ICAC prevented this effect. This finding was further validated in an LPS-induced inflammatory model in RAW 264.7 cells, where ICAC similarly preserved ΔΨ_M_. Additionally, EtOH induced both intracellular and mitochondrial oxidative stress in both AGS and RAW 264.7 cells, while ICAC alleviated oxidative stress in both cell types. These results support the hypothesis that oxidative stress is a central contributor to gastritis pathogenesis and demonstrate that ICAC exerts significant antioxidant effects.

Our findings also suggest that oxidative stress-induced gastritis is mediated through the phosphorylation of DRP1 S616. This mechanism is supported by prior studies showing that DRP1-mediated mitochondrial fission enhances oxidative stress and apoptosis [27] and that DRP1- and mitofusin-mediated oxidative stress contribute to cellular and mitochondrial dysfunction [28]. Additionally, Wang *et al*. [29] showed that urban particulate matter induced DRP1 S616 phosphorylation and mitochondrial fission, contributing to endothelial cell injury. Consistent with these studies, we demonstrated that EtOH rapidly induced DRP1 S616 phosphorylation and that this increase was effectively suppressed by ICAC in both AGS and RAW 264.7 cells. These observations implicate DRP1-driven mitochondrial fragmentation as a key mechanism in EtOH-induced gastric injury.

To further investigate cellular signaling events, we examined the roles of Ca^2+^-mediated pathways in mitochondrial fission during gastritis. The mitochondrial Ca^2+^ transporter MCU is critical for maintaining Ca^2+^ homeostasis and mitochondrial function [30]. Notably, elevated MCU expression has been reported in gastric cancer and is associated with poor clinical outcomes due to its impact on oxidative phosphorylation, the NAD^+^–NADH balance, the tricarboxylic acid cycle, and overall metabolic function [31]. In cardiac myocytes, Hom *et al*.[32] also reported that Ca^2+^ signaling activates DRP1 and induces ROS production. In line with these findings, the current study showed that EtOH increased mitochondrial Ca^2+^ influx and MCU expression, while ICAC effectively suppressed these changes. These findings suggest that Ca^2+^-mediated signaling contributes to EtOH-induced mitochondrial dysfunction and that ICAC can protect mitochondrial integrity by modulating mitochondrial Ca^2+^ transport.

Since previous studies on chlorogenic acid and caffeic acid derivatives have primarily focused on their anti-inflammatory effects in cell lines or the phenotypic improvements to gastritis they produce in mouse models [33, 34], the cellular mechanism underlying how such compounds alleviate gastritis remains poorly understood. Fully characterizing this mechanistic basis is essential for therapeutic development. In this study, we demonstrated that ICAC exerts anti-gastritis effects comparable to those of chlorogenic acid [33], and further, that it prevents mucosal damage and mitochondrial dysfunction in a mouse model and newly established human gastric cell models. Our findings suggest that ICAC possesses great potential as a therapeutic candidate for gastritis and provide new insights for future drug development.

To validate the therapeutic relevance of ICAC in a natural formulation, we examined the effects of EAA, an extract enriched in ICAC. We found that EAA prevented EtOH–HCl-induced acute gastritis in the mouse model. It attenuated inflammatory responses in mouse gastric tissue, demonstrating the potential of natural products containing ICAC as promising candidates for gastritis treatment.

The *in vitro* model used in this study offers a practical and reproducible system for screening gastroprotective agents. Unlike current treatments that primarily focus on symptom management, our approach focuses on mechanistic insights and provides a platform for identifying compounds that target the root causes of gastritis. Nevertheless, further *in vivo* validation and clinical studies are necessary to confirm the translational potential and therapeutic efficacy of this model and the identified candidate materials.

## Conclusion

This study confirmed that EtOH induces acute gastritis by damaging the gastric mucosa and impairing mitochondrial function through Ca^2+^-mediated mitochondrial fission and oxidative stress. To counteract these detrimental effects, we demonstrated the protective efficacy of ICAC in both *in vivo* and *in vitro* models. Isochlorogenic acid C preserved gastric mucosal homeostasis by modulating the PTGS1–PGE_2_ signaling axis and alleviated mitochondrial dysfunction by regulating Ca^2+^-mediated mitochondrial fission, oxidative stress, and ΔΨ_M_. Furthermore, the oral administration of EAA, which consists primarily of ICAC, significantly alleviated EtOH–HCl-induced acute gastritis in mice. Collectively, our findings suggest that ICAC is a promising nutraceutical candidate for the prevention and management of gastrointestinal diseases.

## Supplemental Materials

Supplementary data for this paper are available on-line only at http://jmb.or.kr.



## References

[1] Kayacetin S, Guresci S. 2014. What is gastritis? What is gastropathy? How is it classified? *Turk. J. Gastroenterol.* **25:** 233-247. 10.5152/tjg.2014.7906 25141310

[2] Zuzek R, Potter M, Talley NJ, Agreus L, Andreasson A, Veits L (2024). Prevalence of histological gastritis in a community population and association with epigastric pain. Dig. Dis. Sci..

[3] Sugano K, Tack J, Kuipers EJ, Graham DY, El-Omar EM, Miura S (2015). Kyoto global consensus report on *Helicobacter pylori* gastritis. Gut.

[4] Bode C, Bode JC (1997). Alcohol's role in gastrointestinal tract disorders. Alcohol Health Res. World.

[5] Feyisa ZT, Woldeamanuel BT (2021). Prevalence and associated risk factors of gastritis among patients visiting Saint Paul Hospital Millennium Medical College, Addis Ababa, Ethiopia. PLoS One.

[6] Neutel CI, Appel WC (2000). The effect of alcohol abuse on the risk of NSAID-related gastrointestinal events. Ann. Epidemiol..

[7] Wilson DE (1991). Role of prostaglandins in gastroduodenal mucosal protection. J. Clin. Gastroenterol..

[8] Miller TA (1983). Protective effects of prostaglandins against gastric mucosal damage: current knowledge and proposed mechanisms. Am. J. Physiol..

[9] Takeuchi K, Amagase K (2018). Roles of cyclooxygenase, prostaglandin E2 and EP receptors in mucosal protection and ulcer healing in the gastrointestinal tract. Curr. Pharm. Des..

[10] Chen W, Zhao H, Li Y (2023). Mitochondrial dynamics in health and disease: mechanisms and potential targets. Signal Transduct. Target. Ther..

[11] Weinberg SE, Sena LA, Chandel NS (2015). Mitochondria in the regulation of innate and adaptive immunity. Immunity.

[12] Picard M, Shirihai OS (2022). Mitochondrial signal transduction. Cell Metab..

[13] Chandel NS (2014). Mitochondria as signaling organelles. BMC Biol..

[14] Berridge MJ (2012). Calcium signalling remodelling and disease. Biochem. Soc. Trans..

[15] Peng TI, Jou MJ (2010). Oxidative stress caused by mitochondrial calcium overload. Ann. N Y Acad. Sci..

[16] Pan JS, He SZ, Xu HZ, Zhan XJ, Yang XN, Xiao HM (2008). Oxidative stress disturbs energy metabolism of mitochondria in ethanol-induced gastric mucosa injury. World J. Gastroenterol..

[17] Puangpraphant S, Berhow MA, Vermillion K, Potts G, Gonzalez de Mejia E (2011). Dicaffeoylquinic acids in Yerba mate (*Ilex paraguariensis* St. Hilaire) inhibit NF-kappaB nucleus translocation in macrophages and induce apoptosis by activating caspases-8 and -3 in human colon cancer cells. Mol. Nutr. Food Res..

[18] Urushisaki T, Takemura T, Tazawa S, Fukuoka M, Hosokawa-Muto J, Araki Y (2011). Caffeoylquinic acids are major constituents with potent anti-influenza effects in brazilian green propolis water extract. Evid. Based Complement. Alternat. Med..

[19] Choi J, Park JK, Lee KT, Park KK, Kim WB, Lee JH (2005). *In vivo* antihepatotoxic effects of Ligularia fischeri var. spiciformis and the identification of the active component, 3,4-dicaffeoylquinic acid. J. Med. Food.

[20] Han EH, Kim JY, Kim HG, Chun HK, Chung YC, Jeong HG (2010). Inhibitory effect of 3-caffeoyl-4-dicaffeoylquinic acid from *Salicornia herbacea* against phorbol ester-induced cyclooxygenase-2 expression in macrophages. Chem. Biol. Interact..

[21] Chun A, Paik SJ, Park J, Kim R, Park S, Jung SK (2023). Physicochemical and functional properties of yeast-fermented cabbage. J. Microbiol. Biotechnol..

[22] Paik SJ, Kim DS, Son JE, Bach TT, Hai DV, Paik JH (2024). Validation of active compound of *Terminalia catappa* L. extract and its anti-inflammatory and antioxidant properties by regulating mitochondrial dysfunction and cellular signaling pathways. J. Microbiol. Biotechnol..

[23] Kapur BM, Lala PK, Shaw JL (2014). Pharmacogenetics of chronic pain management. Clin. Biochem..

[24] Reis CA, David L, Correa P, Carneiro F, de Bolos C, Garcia E (1999). Intestinal metaplasia of human stomach displays distinct patterns of mucin (MUC1, MUC2, MUC5AC, and MUC6) expression. Cancer Res..

[25] Boulton DP, Caino MC (2022). Mitochondrial fission and fusion in tumor progression to metastasis. Front. Cell. Dev. Biol..

[26] Drummond RM, Mix TC, Tuft RA, Walsh JV, Jr., Fay FS. 2000. Mitochondrial Ca^2+^ homeostasis during Ca^2+^ influx and Ca^2+^ release in gastric myocytes from Bufo marinus. *J. Physiol.* **522 Pt 3**: 375-390. 10.1111/j.1469-7793.2000.t01-2-00375.x 10713963 PMC2269764

[27] Chang X, Niu S, Shang M, Li J, Guo M, Zhang W (2023). ROS-Drp1-mediated mitochondria fission contributes to hippocampal HT22 cell apoptosis induced by silver nanoparticles. Redox Biol..

[28] Wu S, Zhou F, Zhang Z, Xing D (2011). Mitochondrial oxidative stress causes mitochondrial fragmentation via differential modulation of mitochondrial fission-fusion proteins. FEBS J..

[29] Wang Y, Kong L, Wu T, Tang M (2020). Urban particulate matter disturbs the equilibrium of mitochondrial dynamics and biogenesis in human vascular endothelial cells. Environ. Pollut..

[30] Fan M, Zhang J, Tsai CW, Orlando BJ, Rodriguez M, Xu Y (2020). Structure and mechanism of the mitochondrial Ca^2+^ uniporter holocomplex. Nature.

[31] Xu Z, Chen X, Zhou H, Sun L, Bai R, Yu W (2024). The clinical significance of mitochondrial calcium uniporter in gastric cancer patients and its preliminary exploration of the impact on mitochondrial function and metabolism. Front. Oncol..

[32] Hom J, Yu T, Yoon Y, Porter G, Sheu SS (2010). Regulation of mitochondrial fission by intracellular Ca^2+^ in rat ventricular myocytes. Biochim. Biophys. Acta.

[33] Shimoyama AT, Santin JR, Machado ID, de Oliveira e Silva AM, de Melo ILP (2012). Antiulcerogenic activity of chlorogenic acid in different models of gastric ulcer. Naunyn Schmiedebergs Arch. Pharmacol..

[34] Marengo A, Fumagalli M, Sanna C, Maxia A, Piazza S, Cagliero C (2018). The hydro-alcoholic extracts of Sardinian wild thistles (*Onopordum* spp.) inhibit TNFα-induced IL-8 secretion and NF-κB pathway in human gastric epithelial AGS cells. J. Ethnopharmacol..

