# Supplementary material for: Isochlorogenic Acid C and Artemisia argyi Extract Prevent Acute Gastritis by Mitigating Gastric Mucosa Injury and Mitochondrial Dysfunction
Source: J Microbiol Biotechnol. 2025 Dec 18;35:e2511015. doi: 10.4014/jmb.2511.11015 (PMC12740846; doi:10.4014/jmb.2511.11015)
Supplement: Supplementary file 1 [file jmb-35-e2511015-supple.pdf]

**J. Microbiol. Biotechnol. 2025. 35: e2511015**

https://doi.org/10.4014/jmb.2511.11015

## Supplementary Table and Figure

# Isochlorogenic Acid C and *Artemisia argyi* Extract Prevent Acute Gastritis by Mitigating Gastric Mucosa Injury and Mitochondrial Dysfunction

**So Jeong Paik[1], Hai-Hua Jiang[2], Sang-Ho Lee[2], Eun-Hye Han[2], Na-Young Yun[2] and Sung Keun Jung[1,3*]**

[1] School of Food Science and Biotechnology, Kyungpook National University, Daegu, Republic of Korea

[2] Research Center, Dong-A Pharm. Co., Ltd., Yongin 17073, Republic of Korea

[3] Research Institute of Tailored Food Technology, Kyungpook National University, Daegu, Republic of Korea

[*] Corresponding author: skjung04@knu.ac.kr

**Table S1. Sequences and target genes of the primers used for qRT-PCR in this study.**

| Origin | Gene | Sequences | |
|---|---|---|---|
| Mouse | *Muc1* | Forward | GGG TCT CCT TCT TCT TCT TGT C |
| | | Reverse | GTT CCT CTT CAG TTC TTG GTA GT |
| | *Muc5ac* | Forward | CGA TGT GTA GCC AGG ATT GT |
| | | Reverse | GTG GCG TGG TAG ATG TAG ATA G |
| | *Muc6* | Forward | CAC ACA GCA CAC ACC CTA TAT C |
| | | Reverse | GGA TGG TGT TGG GAA GGT AAT C |
| | *Ptgs1* | Forward | AAG ATG GGT CCT GGC TTT AC |
| | | Reverse | GGT GAT ACT GTC GTT CCA GAT T |
| Human | *PTGS1* | Forward | CAC TTC ACC CAC CAG TTC TT |
| | | Reverse | CGC TCC AGA TTG TCT CCA TAA A |

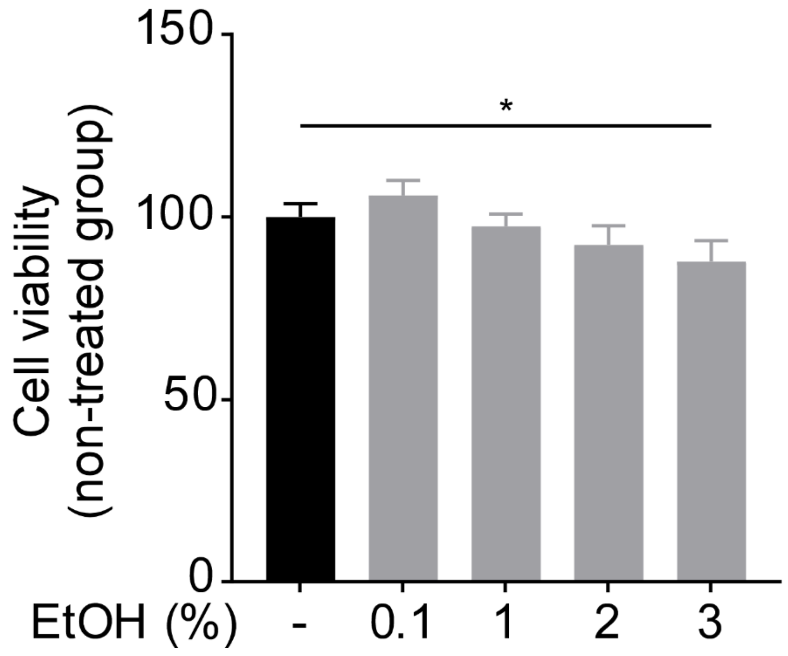


**Fig. S1. The effect of different concentrations of ethanol (EtOH) on the cell viability of AGS cells.** Cell viability was detected using the MTT assay. Statistical significance was analyzed using an one-way ANOVA followed by Dunnett's *post hoc* test. The asterisk, *, indicates a significant difference ($p < 0.05$) in cell viability between the cells treated with 3% EtOH and the non-treated cells.
